# Disparities in Human Papillomavirus–Associated Cancer Incidence by Appalachian Residence

**DOI:** 10.1001/jamanetworkopen.2025.18242

**Published:** 2025-06-30

**Authors:** Todd Burus, Melina J. Windon, Yasminka A. Jakubek, Krystle A. Lang Kuhs

**Affiliations:** 1 Markey Cancer Center, University of Kentucky, Lexington; 2 Department of Otolaryngology–Head & Neck Surgery, College of Medicine, University of Kentucky, Lexington; 3 Department of Internal Medicine, College of Medicine, University of Kentucky, Lexington; 4 Department of Epidemiology & Environmental Health, College of Public Health, University of Kentucky, Lexington

## Abstract

**Importance:**

Poor socioeconomic conditions and corresponding health disparities have historically characterized the Appalachian region of the US. Low uptake of the human papillomavirus (HPV) vaccine and high rates of cervical cancer have been observed in the region; however, a comprehensive assessment of HPV-associated cancer in Appalachia has not been performed.

**Objective:**

To compare the burden of HPV-associated cancer incidence between the Appalachian and non-Appalachian regions of the US.

**Design, Setting, and Participants:**

This cross-sectional study of HPV-associated cancer incidence rates and trends used the US Cancer Statistics Incidence Analytic Database, covering 99% of the US population and 100% of the Appalachian region. Participants were individuals with an HPV-associated cancer diagnosis reported to a US cancer registry between January 1, 2004, and December 31, 2021. Human papillomavirus–associated cancers include squamous cell carcinomas of the oropharynx, anus, vulva, vagina or penis, or cervical carcinoma. Statistical analysis was performed in December 2024.

**Exposures:**

Age, sex, race and ethnicity, county of residence, urbanicity, and stage at diagnosis.

**Main Outcomes and Measures:**

Estimated HPV-associated cancer incidence rates for 2017 to 2021 and annual incidence rate trends between 2004 and 2021. Calculated incidence rate ratios (IRRs) and differences in average annual percentage change by Appalachian residence. Additional comparisons were made between Appalachian regions.

**Results:**

From 2017 to 2021, there were 23 649 cases of HPV-associated cancer diagnosed among Appalachian residents (12 929 females [54.7%]). The overall HPV-associated cancer incidence was 16% higher among Appalachian residents than non-Appalachian residents (IRR, 1.16; 95% CI, 1.14-1.18). Higher site-specific rates among Appalachian residents were noted for all HPV-associated cancer sites, except vaginal cancer and male anal cancer, with the greatest disparity occurring for vulvar cancer (IRR, 1.44; 95% CI, 1.38-1.51). Human papillomavirus–associated cancer incidence increased significantly faster in Appalachia vs non-Appalachia between 2004 and 2021 (average annual percentage change, 1.3% per year [95% CI, 1.0%-1.6% per year]; vs 0.7% per year [95% CI, 0.4%-1.0% per year]; *P* = .004), with the most marked trend difference occurring for penile cancer (2.1% faster per year in Appalachia; *P* = .003). Within Appalachia, the highest HPV-associated incidence rates per 100 000 persons were observed in the North Central (16.9 [95% CI, 16.2-17.6]) and Central (16.9 [95% CI, 16.1-17.7]) subregions.

**Conclusions and Relevance:**

This cross-sectional study of HPV-associated cancer incidence found disproportionately high HPV-associated cancer rates among Appalachian residents compared with non-Appalachian residents. These findings highlight the need for targeted efforts to improve HPV vaccine uptake and encourage adherence to evidence-based screening guidelines for HPV-associated cancers in Appalachia.

## Key Points

**Question:**

Do differences exist in the incidence of human papillomavirus (HPV)–associated cancers between the Appalachian and non-Appalachian regions of the US?

**Findings:**

This population-based cross-sectional study found that HPV-associated cancer incidence from 2017 to 2021 was 16% higher among individuals in Appalachia than among those living outside Appalachia. Site-specific disparities were seen for HPV-associated male and female oropharyngeal cancers, female anal cancer, vulvar cancer, cervical cancer, and penile cancer.

**Meaning:**

This study suggests that the Appalachian region experiences a greater burden of HPV-associated cancer incidence than elsewhere in the US.

## Teaser

This cross-sectional study uses a dataset covering 100% of the Appalachian region to compare the burden of human papillomavirus–associated cancer incidence between the Appalachian and non-Appalachian regions of the US.

## Introduction

Human papillomavirus (HPV) is associated with the development of certain cancers of the oropharynx, anus and rectum, vulva, vagina, cervix, and penis.^1,2^ Although several highly effective HPV vaccines are available, low uptake among certain populations threatens to moderate their effect. Thus, it is crucial to identify geographic regions with higher HPV-associated cancer burden to inform targeted outreach and vaccination efforts.

The Appalachian region of the US is a federally recognized collection of 423 counties spanning 13 eastern states.^3^ This region is historically connected with poor socioeconomic conditions and experiences worse health outcomes when compared with the rest of the US.^4,5,6,7^ Previous studies have reported Appalachian disparities in HPV-associated cancer incidence^8,9,10,11,12^; however, these studies were often limited in scope, restricted to specific cancer sites or geographic regions within Appalachia. One study examining all HPV-associated cancer sites, but only using data from Ohio, Kentucky, and West Virginia, found Appalachian disparities in penile, vulvar, and cervical cancer.^8^ Several other studies have also reported disparities in cervical cancer incidence, including a study by Damgacioglu et al,^9^ which found significantly faster increases in the rates of late-stage cervical cancer incidence in Appalachian vs non-Appalachian Kentucky.^10,11^ Higher rates of HPV-associated penile cancer have also been reported in Appalachian Kentucky.^12^ Using a dataset covering 100% of the Appalachian region, we conducted the first comprehensive study, to our knowledge, of the burden of HPV-associated cancers in Appalachia.

## Methods

This study was deemed exempt from review and informed consent by the University of Kentucky institutional review board due to the use of deidentified data. We followed the Strengthening the Reporting of Observational Studies in Epidemiology (STROBE) reporting guideline.

### Data Sources

We calculated rates of incident cancers between January 1, 2004, and December 31, 2021, from the US Cancer Statistics (USCS) Incidence Analytic Database, 1998-2021.^13^ This database contains records from the Centers for Disease Control and Prevention (CDC) National Program of Cancer Registries and the National Cancer Institute Surveillance, Epidemiology, and End Results (SEER) program. Cases from before 2004 were excluded to provide consistent coverage of stage at diagnosis variables. In addition, cases from Indiana were excluded due to not meeting USCS publication criteria for 2020 and 2021. The USCS Incidence Analytic Database covers more than 99% of the US population during the study period and 100% of the Appalachian region.

### Study Design

We used *International Classification of Diseases for Oncology, Third Edition* morphology codes to identify HPV-associated cancer sites consistent with definitions provided by USCS.^14^ Cancer sites considered HPV-associated were oropharyngeal squamous cell carcinoma, anal and rectal squamous cell carcinoma, vulvar squamous cell carcinoma, vaginal squamous cell carcinoma, cervical carcinoma, and penile squamous cell carcinoma. Specific site and histologic codes used can be found in eTable 1 in Supplement 1. We included only malignant and microscopically confirmed cases. Stage at diagnosis was determined using the Merged Summary Stage variable. Localized cases were classified as early stage, while cases with regional and distant metastasis were classified as late stage.

**Figure 1.**
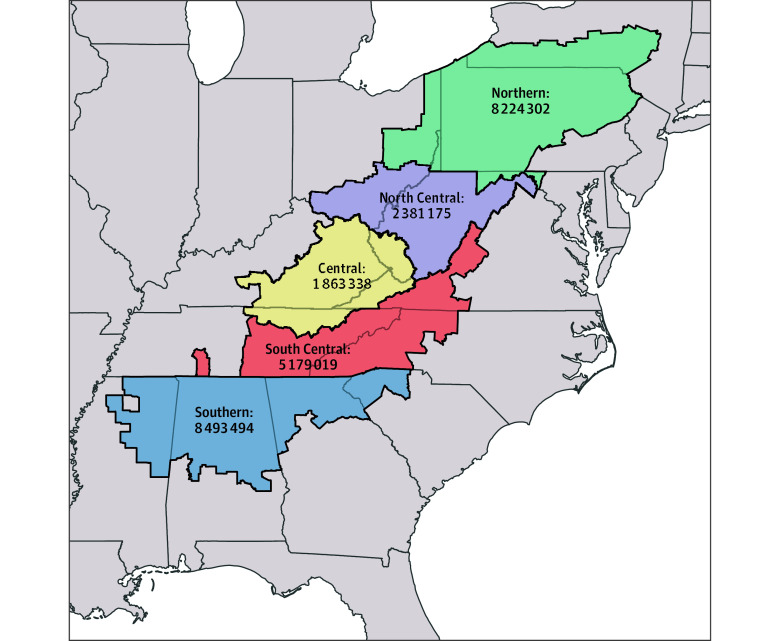
Map of Appalachian Subregions With Population Estimates, Appalachian Regional Commission and US Census Bureau American Community Survey 5-Year Estimates, 2017-2021^3,15^

We analyzed patient demographics according to Appalachian residence, sex, race and ethnicity, and urbanicity. Appalachian residence was defined by county of residence at time of diagnosis following classification developed by the Appalachian Regional Commission.^3^ Additional classification into Appalachian subregion was applied to certain analyses (Figure 1).^3,15^ We classified race and ethnicity into Hispanic (all races), non-Hispanic Black, non-Hispanic White, and other non-Hispanic races according to the USCS race and origin recode variable. Other non-Hispanic races includes individuals identified as non-Hispanic American Indian or Alaska Native, non-Hispanic Asian or Pacific Islander, and non-Hispanic unknown race. Race and ethnicity information in the USCS database is extracted from medical records by cancer registry personnel and was included to assess whether racial or ethnic disparities in HPV-associated cancer incidence were present in Appalachia. We used 2013 Rural-Urban Continuum Codes (RUCCs) for the county of residence at time of diagnosis to determine urbanicity, with RUCCs 1 to 3 classified as metropolitan and RUCCs 4 to 9 classified as nonmetropolitan.^16^

### Statistical Analysis

Statistical analysis was performed in December 2024. We collected 5-year incidence rates for 2017-2021 for combined and site-specific HPV-associated cancers, stratified by Appalachian residence and sex, race and ethnicity, urbanicity, and/or stage at diagnosis. Additional annual incidence rates were collected for 2004 to 2021, stratified by Appalachian residence and sex and stage at diagnosis. All incidence rates were calculated using SEER*Stat, version 8.4.3 (Surveillance Research Program, National Cancer Institute), and age-adjusted per 100 000 persons to the 2000 US standard population. Rates were suppressed for any population subgroups containing fewer than 16 cases.

We used incidence rate ratios (IRRs) to compare rates between population subgroups. The Tiwari method was used to calculate IRRs with corresponding 95% CIs. Temporal trends in annual incidence rates were assessed by fitting piecewise log-linear regression models to the data using Joinpoint Software, version 5.2.0 (Statistical Methodology and Applications Branch, Surveillance Research Program, National Cancer Institute). Trends were used to estimate average annual percentage change with 95% CIs. We excluded rates for 2020 from trend analysis due to documented disruptions in the diagnosis of new cancers during the COVID-19 pandemic.^17^

Statistical significance was assessed at a level of *P* < .05, and all hypotheses were 2-sided. All additional analyses were performed using R, version 4.3.3 (R Project for Statistical Computing).

## Results

### Appalachian HPV-Associated Cancer Incidence, 2017-2021

Between 2017 and 2021, there were 23 649 new cases of HPV-associated cancer diagnosed among residents of Appalachia (12 929 females [54.7%] and 10 720 males [45.3%]; 1354 non-Hispanic Black residents [5.7%], 376 Hispanic residents [1.6%], 21 630 non-Hispanic White residents [91.5%]; and 289 residents of other race or ethnicity [1.2%]) (Table 1).^13^ Human papillomavirus–associated cancers were diagnosed most frequently among females (12 929 cases [54.7%]), persons of non-Hispanic White race and ethnicity (21 630 cases [91.5%]), and individuals living in metropolitan counties (14 972 cases [63.3%]). Cervical cancer accounted for the most diagnoses among females (5267 of 12 929 cases [40.7%]), while oropharyngeal cancer was most frequent for males (8868 of 10 720 cases [82.7%]).

**Table 1.** Human Papillomavirus–Associated Cancer Incidence by Appalachian Residence and Selected Characteristics, US Cancer Statistics^13^

Characteristic	Appalachia		Non-Appalachia	
	Cases, No. (%)	Incidence rate (95% CI)^a^	Cases, No. (%)	Incidence rate (95% CI)
Overall	23 649	14.3 (14.1-14.5)	216 271	12.4 (12.3-12.4)
Sex				
Male	10 720 (45.3)	12.5 (12.2-12.7)	97 797 (45.2)	11.1 (11.0-11.1)
Female	12 929 (54.7)	16.2 (15.9-16.5)	118 474 (54.8)	13.7 (13.7-13.8)
Race and ethnicity				
Hispanic (all races)	376 (1.6)	7.9 (7.1-8.9)	24 133 (11.2)	10.3 (10.1-10.4)
Non-Hispanic Black	1354 (5.7)	10.1 (9.5-10.7)	22 429 (10.4)	11.2 (11.1-11.4)
Non-Hispanic White	21 630 (91.5)	15.2 (14.9-15.4)	160 618 (74.3)	13.5 (13.4-13.5)
Other non-Hispanic race^a^	289 (1.2)	9.4 (8.3-10.6)	9091 (4.2)	7.5 (7.4-7.7)
Urbanicity				
Metropolitan	14 972 (63.3)	13.8 (13.6-14.1)	182 902 (84.6)	12.1 (12.1-12.2)
Nonmetropolitan	8677 (36.7)	15.4 (15.0-15.7)	31 104 (14.4)	13.9 (13.8-14.1)
Unknown	0	No data	2265 (1.0)	No data
Site				
Oropharyngeal				
Female	1913 (8.1)	2.0 (1.9-2.1)	16 295 (7.5)	1.7 (1.6-1.7)
Male	8868 (37.5)	10.1 (9.9-10.3)	80 290 (37.1)	8.9 (8.9-9.0)
Anal				
Female	2690 (11.4)	3.0 (2.8-3.1)	24 127 (11.2)	2.5 (2.5-2.5)
Male	1067 (4.5)	1.3 (1.3-1.4)	11 388 (5.3)	1.4 (1.3-1.4)
Vulvar	2646 (11.2)	3.0 (2.9-3.1)	19 445 (9.0)	2.1 (2.0-2.1)
Vaginal	413 (1.7)	0.5 (0.4-0.5)	4077 (1.9)	0.4 (0.4-0.4)
Cervical	5267 (22.3)	7.8 (7.6-8.0)	54 530 (25.2)	7.1 (7.0-7.2)
Penile	785 (3.3)	1.0 (0.9-1.1)	6119 (2.8)	0.8 (0.7-0.8)

^a^ Rate age-adjusted according to 2000 US standard population and reported per 100 000 residents.

**Figure 2.**
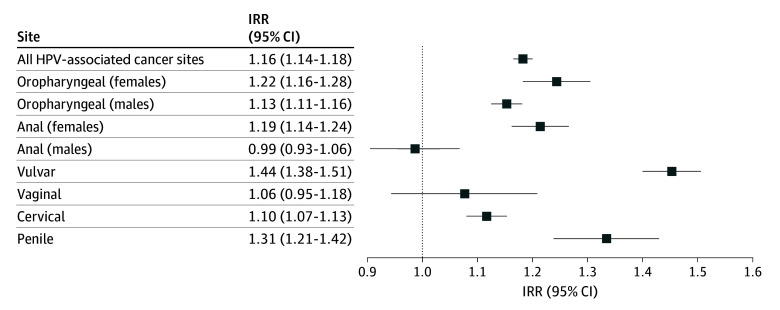
Incidence Rate Ratios (IRRs) for Human Papillomavirus (HPV)–Associated Cancer Incidence, Appalachia vs Non-Appalachia, 2017-2021, US Cancer Statistics^13^ Ratio of incidence rate for Appalachian individuals to incidence rate for non-Appalachian individuals for all HPV-associated cancer sites combined and 6 sites individually. Incidence rate ratios significantly different than 1 if 1 is not included in the 95% CI.

Overall, HPV-associated cancers had an age-adjusted incidence rate of 14.3 cases (95% CI, 14.1-14.5 cases) per 100 000 persons residing in Appalachia (Table 1).^13^ This was a significant 16% higher than among non-Appalachian individuals (incidence rate per 100 000 persons, 12.4 [95% CI, 12.3-12.4]; IRR, 1.16; 95% CI, 1.14-1.18) (Figure 2).^13^ Human papillomavirus–associated cancer incidence among Appalachian females (incidence rate per 100 000 persons, 16.2 [95% CI, 15.9-16.5]) was 18% higher than among non-Appalachian females (incidence rate per 100 000 persons, 13.7 [95% CI, 13.7-13.8]; IRR, 1.18 [95% CI, 1.16-1.20]) and HPV-associated cancer incidence among Appalachian males (incidence rate per 100 000 persons, 12.5 [95% CI, 12.2-12.7]) was 13% higher than among non-Appalachian males (incidence rate per 100 000 persons, 11.1 [95% CI, 11.0-11.1]; IRR, 1.13; [95% CI, 1.10-1.15]) (Table 1).^13^ Site-specific incidence rates by sex were also significantly higher among persons in Appalachia for all sites considered except for male anal cancer and vaginal cancer. The largest difference in incidence was observed for vulvar cancer, where rates among Appalachian females (incidence rate per 100 000 persons, 3.0; 95% CI, 2.9-3.1) were 44% higher than among non-Appalachian females (incidence rate per 100 000 persons, 2.1 [95% CI, 2.0-2.1]; IRR, 1.44 [95% CI, 1.38-1.51]). Diagnosis of late-stage HPV-associated cancer occurred at a 17% higher rate among residents of Appalachia (IRR, 1.17 [95% CI, 1.15-1.19]), with all except male anal cancer having significantly higher site-specific rates of late-stage diagnosis by sex (eTable 2 in Supplement 1).

### Trends in Appalachian HPV-Associated Cancer Incidence, 2004-2021

**Figure 3.**
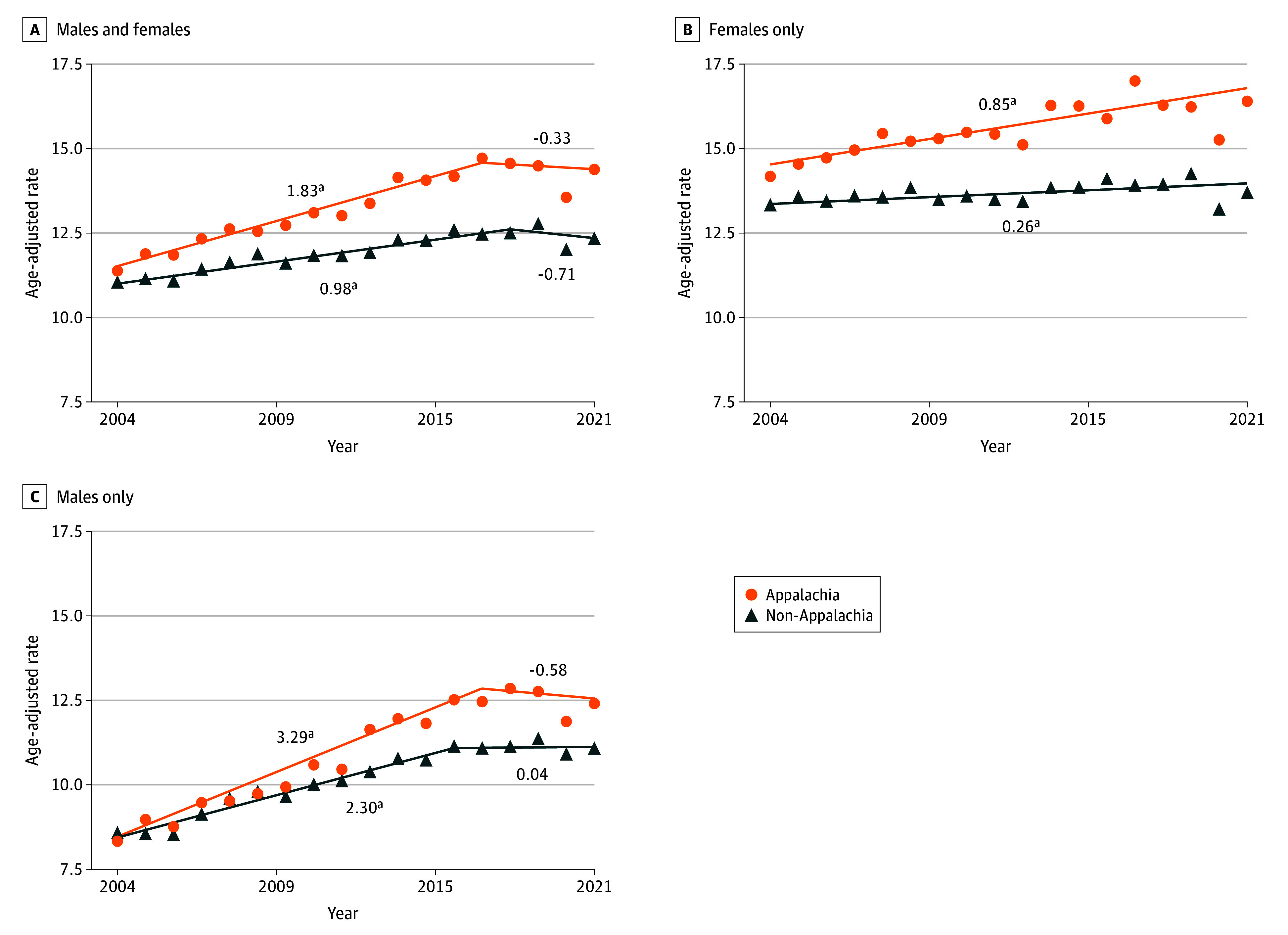
Trends in Human Papillomavirus (HPV)–Associated Cancer Incidence Rates by Appalachian Residence, 2004-2021, US Cancer Statistics^13^ Incidence rates for 2004 to 2021 with fitted joinpoint regression model and segment annual percentage change shown. Rates for 2020 were excluded from models due to the association of the COVID-19 pandemic with cancer diagnoses. ^a^Significant annual percentage change.

Human papillomavirus–associated cancer incidence among Appalachian residents increased by an average annual percentage change of 1.3% (95% CI, 1.0%-1.6%) per year between 2004 and 2021, significantly faster than the increase of 0.7% (95% CI, 0.4%-1.0%) per year outside of Appalachia (*P* = .004) (Figure 3).^13^ This included a 0.6%-per-year faster increase for Appalachian females compared with non-Appalachian females (*P* < .001), and a 0.7%-per-year faster increase for Appalachian males compared with non-Appalachian males (*P* = .03). Significantly faster increases occurred in Appalachia vs non-Appalachia for male oropharyngeal cancer (0.7% faster per year; *P* = .04), female oropharyngeal cancer (1.5% faster per year; *P* = .02), and penile cancer (2.1% faster per year; *P* = .003) (eFigure 1 and eTable 3 in Supplement 1). No HPV-associated cancer site had significantly decreasing sex-specific incidence rates in either Appalachia or non-Appalachia between 2004 and 2021.

Late-stage HPV-associated cancer diagnoses in Appalachia increased 0.8% per year faster than in non-Appalachia (*P* = .006; eTable 4 in Supplement 1). Increases in late-stage penile cancer diagnoses were observed among males in both regions, with rates among Appalachian males increasing more than twice as fast as rates among non-Appalachian males (2.4% per year faster; *P* = .004).

### Disparities in HPV-Associated Cancer Incidence Within the Appalachian Region

Within the Appalachian region, incidence rates of HPV-associated cancers were 11% higher among residents of nonmetropolitan counties (8 717 975 of 26 154 834 people, or 33.3% of study population) compared with metropolitan counties (IRR, 1.11; 95% CI, 1.08-1.14) (eTable 5 in Supplement 1), and highest among non-Hispanic White individuals (incidence rate per 100 000 persons, 15.2 [95% CI, 14.9-15.4) (eTables 5 and 6 in Supplement 1).

Substantial geographic variation in HPV-associated cancer incidence was also observed by Appalachian subregion. Incidence was highest among individuals living in the North Central (incidence rate per 100 000 persons, 16.9 [95% CI, 16.2-17.6]) and Central (incidence rate per 100 000 persons, 16.9 [95% CI, 16.1-17.7]) subregions (Table 2).^13^ The North Central subregion had the highest subregion incidence rates of male oropharyngeal cancer, while the Central and North Central subregions had significantly higher rates of vulvar, cervical, and penile cancers than the other 3 subregions. Overall, HPV-associated cancer incidence rates increased significantly between 2004 and 2021 in every Appalachian subregion except the South Central subregion, with the highest nominal rates in the North Central (2.0% per year) and Central (1.9% per years) subregions (eTable 7 in Supplement 1). Rates of late-stage cervical cancer were highest in the Central subregion (incidence rate per 100 000 persons, 5.8; 95% CI, 5.1-6.6), with a significant increase of 1.7% (95% CI, 0.2%-3.2%) per year between 2004 and 2021 (eTable 8 in Supplement 1). All other subregions experienced stable rates of late-stage cervical cancer during this period (eFigure 2 in Supplement 1).

**Table 2.** HPV-Associated Cancer Incidence by Appalachian Subregion, 2017-2021, US Cancer Statistics^13^

Cancer	Incidence rate (95% CI)				
	Northern	North Central	Central	South Central	Southern
All HPV-associated cancer sites	13.9 (13.6-14.3)	16.9 (16.2-17.6)	16.9 (16.1-17.7)	14.2 (13.8-14.6)	13.4 (13.1-13.8)
Oropharyngeal					
Female	1.9 (1.7-2.1)	2.2 (1.9-2.5)	2.5 (2.1-2.9)	2.2 (2.0-2.5)	1.9 (1.7-2.1)
Male	10.1 (9.7-10.5)	11.6 (10.9-12.4)	10.2 (9.4-11.1)	10.6 (10.1-11.1)	9.3 (8.9-9.7)
Anal					
Female	3.1 (2.9-3.3)	3.7 (3.3-4.2)	3.3 (2.8-3.8)	3.2 (3.0-3.5)	2.4 (2.2-2.6)
Male	1.3 (1.2-1.4)	1.6 (1.3-1.9)	1.3 (1.0-1.7)	1.4 (1.2-1.6)	1.3 (1.1-1.4)
Vulvar	3.1 (2.8-3.3)	3.6 (3.2-4.1)	3.8 (3.3-4.4)	2.7 (2.5-3.0)	2.6 (2.4-2.8)
Vaginal	0.4 (0.3-0.4)	0.4 (0.3-0.6)	0.6 (0.5-0.9)	0.4 (0.3-0.5)	0.5 (0.4-0.6)
Cervical	7.4 (7.0-7.8)	9.1 (8.4-9.9)	10.5 (9.6-11.6)	6.8 (6.4-7.3)	7.8 (7.4-8.2)
Penile	0.8 (0.7-0.9)	1.4 (1.2-1.7)	1.8 (1.5-2.2)	0.9 (0.8-1.1)	1.0 (0.9-1.1)

Abbreviation: HPV, human papillomavirus.

## Discussion

In this cross-sectional study, we found that individuals living in Appalachia had higher, and increasingly worse, incidence rates of HPV-associated cancer than observed among individuals living outside of Appalachia. Results held for both overall diagnoses and late-stage diagnoses. Site-specific disparities among Appalachian individuals compared with non-Appalachian individuals existed for current incidence of all HPV-associated cancers except male anal cancer and vaginal cancer. Disparities also existed in incidence trends for oropharyngeal cancer among both males and females and for penile cancer. Within Appalachia, the HPV-associated cancer burden is concentrated largely in the North Central and Central subregions, containing Appalachian Kentucky, Ohio, and West Virginia.

This study provides, to our knowledge, the first comprehensive analysis of HPV-associated cancer incidence disparities in Appalachia using cancer registry data that cover the entire Appalachian region and incorporates all 6 recognized HPV-associated cancers. This broader perspective offers valuable insights that can help direct public health resources to the areas most in need.

Reasons for the higher HPV-associated cancer burden in Appalachia are unclear, although there are likely several contributing factors. Screening for cervical cancer through the use of Papanicolaou tests and high-risk HPV testing is recommended for all females aged 21 to 65 years in the US.^18^ Such screening provides the opportunity to detect and treat precancerous lesions before they develop into cervical cancer. Consistent data on cervical cancer screening in Appalachia are difficult to obtain due to the lack of an Appalachian indicator in the commonly used Behavioral Risk Factor Surveillance System, but historical analyses have shown lower rates of adherence to cervical cancer screening in the region.^19,20^ Disparities in late-stage cervical cancer incidence among Appalachian populations compared with non-Appalachian populations also suggest screening deficits.^9^ Higher tobacco smoking prevalence in the Appalachian region—particularly in the Central Appalachian subregion—likely plays a role as well, as tobacco smoking has been shown to be associated with increased risk of HPV-associated cancer incidence.^21,22,23^ In addition, high-risk sexual behaviors are prominent in Appalachia, as evidenced by the disproportionately high rate of teen births in the region.^21^

Several evidence-based interventions exist to combat HPV-associated cancer incidence and mortality, although such measures are not currently being used to their full potential in the Appalachian region. Human papillomavirus vaccination has become the cornerstone of primary prevention for HPV-associated cancers in the US since its approval in 2006. However, HPV vaccination uptake in the Appalachian region has been particularly low in certain subregions.^24^ Lack of education about HPV vaccination, poor clinician-to-patient communication, and fears about sexual promiscuity among the youngest eligible individuals have all been identified as barriers to increasing vaccine uptake in Appalachia.^25,26,27,28^ Interventions focused on improving HPV vaccine uptake have been trialed in certain Appalachian communities, but work remains to be done to bring series completion up to CDC target levels across the region.^25,29^ Alongside primary prevention, screening opportunities exist for early detection at some HPV-associated cancer sites. Studies on the barriers to successful cervical cancer screening completion in Appalachia have highlighted fatalistic beliefs about cancer and low clinician availability.^30,31^ Given our findings and the fact that improvements in cervical cancer mortality are largely the result of improved early detection, it is important to study ways to increase adherence to cervical cancer screening, including the use of at-home testing, in the Appalachian region.^32,33^ Ongoing efforts to develop effective screening protocols for HPV-associated oropharyngeal and anal cancers could also prove beneficial at addressing observed Appalachian disparities.^34,35^

### Strengths and Limitations

This study has some strengths, particularly the use of high-quality, population-based cancer registry data covering 100% of the Appalachian region during the study period. This study also has some limitations. First, identification of cancers associated with HPV infection does not entail a causal relationship. However, cancer site is commonly used as a surrogate indicator for HPV association, and using detailed site and histologic definitions from an authoritative source helps maintain consistency with a well-evidenced list of HPV-associated cancers. Second, a more detailed analysis by race and ethnicity is hindered by low racial and ethnic diversity in the Appalachian region. Third, determination of Appalachian residence is subject to misclassification based on patient residence at time of diagnosis.

## Conclusions

In this cross-sectional study of HPV-associated cancer incidence, we found that the Appalachian region of the US had significantly higher rates of HPV-associated cancer than observed among populations outside of Appalachia. Disparities existed both overall and across several specific HPV-associated cancer sites, particularly for oropharyngeal, vulvar, and penile cancers overall, and for late-stage diagnoses of cervical cancer in the Central Appalachian subregion. Increased efforts to improve HPV vaccination rates and adherence to evidence-based cancer screenings are necessary to reduce the disproportionate burden of HPV-associated cancers in Appalachia.

## Notes

Supplement 1. **eTable 1.** Definitions of HPV-Associated Cancers **eTable 2.** Late-Stage Human Papillomavirus (HPV)-Associated Cancer Incidence by Appalachian residence, 2017-2021, US Cancer Statistics **eFigure 1.** Trends in HPV-Associated Cancer Incidence by Site, Sex, and Appalachian Residence, 2004-2021, US Cancer Statistics **eTable 3.** Trends in Human Papillomavirus (HPV)-Associated Cancer Incidence by Appalachian Residence, 2004-2021, US Cancer Statistics **eTable 4.** Trends in Late-Stage Human Papillomavirus (HPV)-Associated Cancer Incidence by Appalachian Residence, 2004-2021, US Cancer Statistics **eTable 5.** Appalachian Human Papillomavirus (HPV)-Associated Cancer Incidence Rates by Urbanicity, 2017-2021, US Cancer Statistics **eTable 6.** Appalachian Human Papillomavirus (HPV)-Associated Cancer Incidence by Race and Ethnicity, 2017-2021, US Cancer Statistics **eTable 7.** Trends in Human Papillomavirus (HPV)-Associated Cancer Incidence by Appalachian Subregion, 2004-2021, US Cancer Statistics **eTable 8.** Trends in Late-Stage Human Papillomavirus (HPV)-Associated Cancer Incidence by Appalachian Subregion, 2004-2021, US Cancer Statistics **eFigure 2.** Trends in Late-Stage Cervical Carcinoma Incidence Rates in Central Appalachia, 2004-2021, US Cancer Statistics
eReferences.

Supplement 2.
Data Sharing Statement
