# Supplementary material for: Disparities in Human Papillomavirus–Associated Cancer Incidence by Appalachian Residence
Source: JAMA Netw Open. 2025 Jun 30;8(6):e2518242. doi: 10.1001/jamanetworkopen.2025.18242 (PMC12210082; doi:10.1001/jamanetworkopen.2025.18242)
Supplement: Supplement 1. — eTable 1. Definitions of HPV-Associated Cancers eTable 2. Late-Stage Human Papillomavirus (HPV)-Associated Cancer Incidence by Appalachian residence, 2017-2021, US Cancer Statistics eFigure 1. Trends in HPV-Associated Cancer Incidence by Site, Sex, and Appalachian Residence, 2004-2021, US Cancer Statistics eTable 3. Trends in Human Papillomavirus (HPV)-Associated Cancer Incidence by Appalachian Residence, 2004-2021, US Cancer Statistics eTable 4. Trends in Late-Stage Human Papillomavirus (HPV)-Associated Cancer Incidence by Appalachian Residence, 2004-2021, US Cancer Statistics eTable 5. Appalachian Human Papillomavirus (HPV)-Associated Cancer Incidence Rates by Urbanicity, 2017-2021, US Cancer Statistics eTable 6. Appalachian Human Papillomavirus (HPV)-Associated Cancer Incidence by Race and Ethnicity, 2017-2021, US Cancer Statistics eTable 7. Trends in Human Papillomavirus (HPV)-Associated Cancer Incidence by Appalachian Subregion, 2004-2021, US Cancer Statistics eTable 8. Trends in Late-Stage Human Papillomavirus (HPV)-Associated Cancer Incidence by Appalachian Subregion, 2004-2021, US Cancer Statistics eFigure 2. Trends in Late-Stage Cervical Carcinoma Incidence Rates in Central Appalachia, 2004-2021, US Cancer Statistics eReferences. [file jamanetwopen-e2518242-s001.pdf]

Supplementary Online Content

Burus T, Windon MJ, Jakubek YA, Lang Kuhs KA. Disparities in human papillomavirus–associated cancer incidence by Appalachian residence. *JAMA Netw Open.* 2025;8(6):e2518242. doi:10.1001/jamanetworkopen.2025.18242

eTable 1. Definitions of HPV-Associated Cancers
eTable 2. Late-Stage Human Papillomavirus (HPV)-Associated Cancer Incidence by Appalachian residence, 2017-2021, US Cancer Statistics
eFigure 1. Trends in HPV-Associated Cancer Incidence by Site, Sex, and Appalachian Residence, 2004-2021, US Cancer Statistics
eTable 3. Trends in Human Papillomavirus (HPV)-Associated Cancer Incidence by Appalachian Residence, 2004-2021, US Cancer Statistics
eTable 4. Trends in Late-Stage Human Papillomavirus (HPV)-Associated Cancer Incidence by Appalachian Residence, 2004-2021, US Cancer Statistics
eTable 5. Appalachian Human Papillomavirus (HPV)-Associated Cancer Incidence Rates by Urbanicity, 2017-2021, US Cancer Statistics
eTable 6. Appalachian Human Papillomavirus (HPV)-Associated Cancer Incidence by Race and Ethnicity, 2017-2021, US Cancer Statistics
eTable 7. Trends in Human Papillomavirus (HPV)-Associated Cancer Incidence by Appalachian Subregion, 2004-2021, US Cancer Statistics
eTable 8. Trends in Late-Stage Human Papillomavirus (HPV)-Associated Cancer Incidence by Appalachian Subregion, 2004-2021, US Cancer Statistics
eFigure 2. Trends in Late-Stage Cervical Carcinoma Incidence Rates in Central Appalachia, 2004-2021, US Cancer Statistics
eReferences.

This supplementary material has been provided by the authors to give readers additional information about their work.

© 2025 Burus T et al. *JAMA Network Open.*

eTable 1. Definitions of HPV-Associated Cancers[1]

Primary site and histology groups were defined using International Classification of Diseases for Oncology version 3 (ICD-0-3).[2] All sites restricted to microscopically confirmed.

Cancer	Primary Sites	Histology Codes
Oropharyngeal squamous cell carcinoma	C01.9, 02.4, 02.8, 05.1-05.2, 09.0–09.1, 09.8–09.9, 10.0–10.4, 10.8–10.9, 14.0, 14.2, 14.8	8050–8086, 8120–8131
Anal and rectal squamous cell carcinoma	C21.0–21.8, 20.9	8050–8084, 8120–8131
Vulvar squamous cell carcinoma	C51.0–51.9	8050–8084, 8120–8131
Vaginal squamous cell carcinoma	C52.9	8050–8084, 8120–8131
Cervical carcinoma	C53.0-53.9	8010–8671, 8940–8941
Penile squamous cell carcinoma	C60.0-60.9	8050–8084, 8120–8131

© 2025 Burus T et al. *JAMA Network Open.*

eTable 2. Late-Stage Human Papillomavirus (HPV)-Associated Cancer Incidence by Appalachian residence, 2017-2021, US Cancer Statistics[5]

**Cancer**	**Appalachia (95% CI)**	**Non-Appalachia (95% CI)**
All HPV-associated	8.97 (8.82, 9.12)	7.65 (7.60, 7.69)
Oropharyngeal (female)	1.57 (1.49, 1.66)	1.27 (1.25, 1.29)
Oropharyngeal (male)	8.60 (8.40, 8.80)	7.51 (7.46, 7.57)
Anal (female)	1.55 (1.46, 1.63)	1.28 (1.25, 1.30)
Anal (male)	0.65 (0.59, 0.71)	0.64 (0.63, 0.66)
Vulvar	0.96 (0.90, 1.03)	0.67 (0.65, 0.68)
Vaginal	0.27 (0.23, 0.30)	0.22 (0.22, 0.23)
Cervical carcinoma	3.98 (3.83, 4.14)	3.51 (3.47, 3.55)
Penile	0.44 (0.39, 0.49)	0.32 (0.31, 0.33)

Abbreviations: CI = Confidence interval

© 2025 Burus T et al. *JAMA Network Open.*

eFigure 1. Trends in HPV-Associated Cancer Incidence by Site, Sex, and Appalachian Residence, 2004-2021, US Cancer Statistics. [9] (A) Oropharyngeal squamous cell carcinoma (SCC), (B) anal and rectal SCC, (C) vulvar SCC, (D) vaginal SCC, (E) cervical carcinoma, and (F) penile SCC. Incidence rates for 2004-2021 with fitted joinpoint regression model and segment annual percentage change shown. Rates for 2020 were excluded from models due to the impact of the COVID-19 pandemic on cancer diagnoses. Significant annual percentage change indicated by (*).

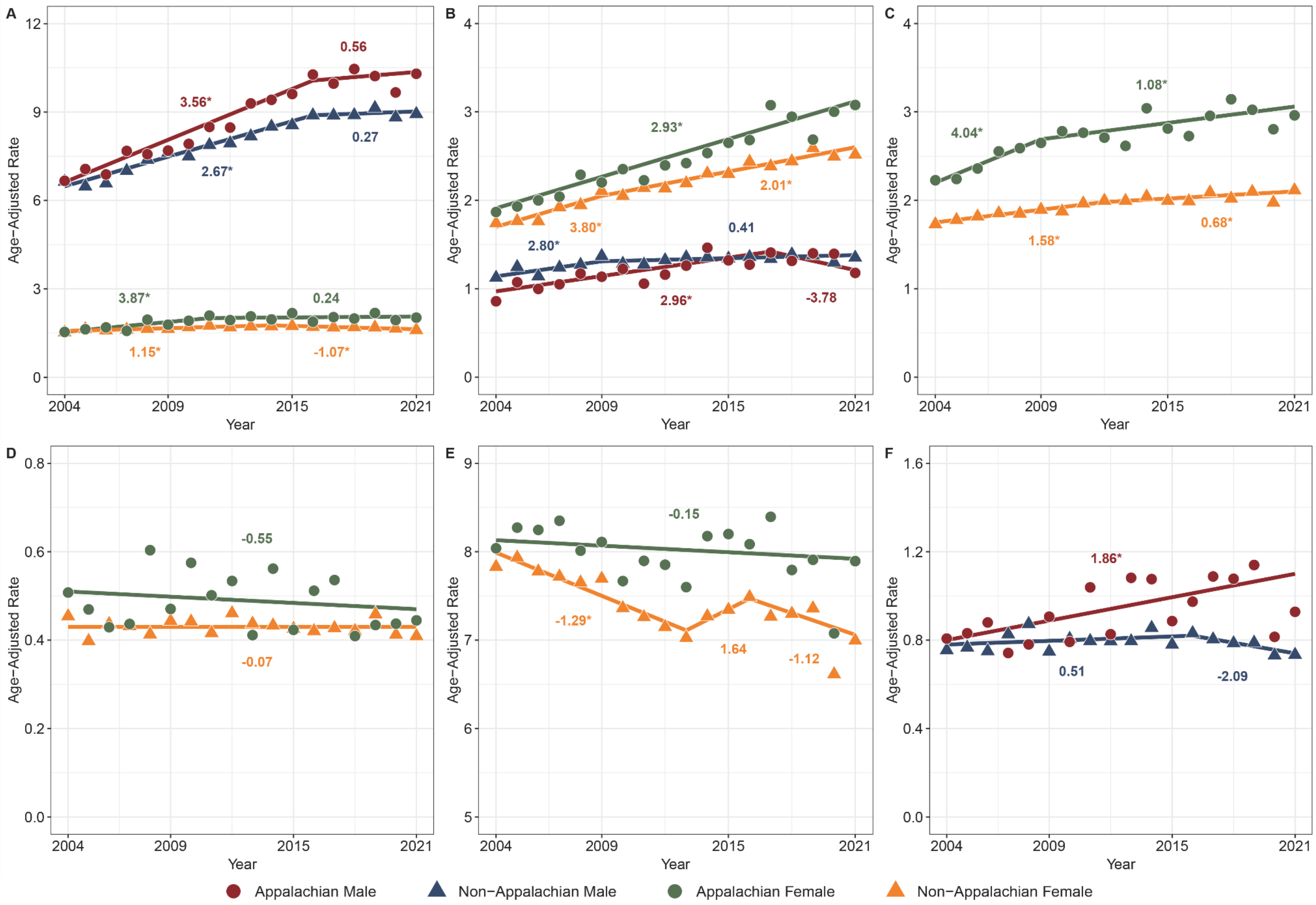

© 2025 Burus T et al. *JAMA Network Open.*

eTable 3. Trends in Human Papillomavirus (HPV)-Associated Cancer Incidence by Appalachian Residence, 2004-2021, US Cancer Statistics[5]

Average annual percentage change (AAPC) between in incidence rates between 2004 and 2021 estimated by joinpoint regression. Rates from 2020 excluded due to disruptions in diagnosis associated with the COVID-19 pandemic.

**Cancer**	**Appalachia (95% CI)**	**Non-Appalachia (95% CI)**	**P-value for Difference**
All HPV-associated	1.32 (1.00, 1.64)	0.68 (0.39, 0.97)	0.004
Oropharyngeal (female)	1.72 (0.54, 2.91)	0.23 (-0.15, 0.61)	0.019
Oropharyngeal (male)	2.67 (2.14, 3.20)	1.96 (1.54, 2.38)	0.039
Anal (female)	2.93 (2.43, 3.44)	2.53 (1.94, 3.14)	0.305
Anal (male)	1.33 (-0.47, 3.17)	1.11 (0.47, 1.75)	0.818
Vulvar	1.94 (0.92, 2.97)	1.10 (0.78, 1.43)	0.126
Vaginal	-0.55 (-1.86, 0.78)	-0.07 (-0.49, 0.36)	0.457
Cervical	-0.15 (-0.45, 0.15)	-0.73 (-1.55, 0.09)	0.188
Penile	1.86 (0.76, 2.98)	-0.27 (-1.24, 0.72)	0.003

Abbreviations: CI = Confidence interval

© 2025 Burus T et al. *JAMA Network Open.*

eTable 4. Trends in Late-Stage Human Papillomavirus (HPV)-Associated Cancer Incidence by Appalachian Residence, 2004-2021, US Cancer Statistics[5]

Average annual percentage change (AAPC) between in incidence rates between 2004 and 2021 estimated by joinpoint regression. Rates from 2020 excluded due to disruptions in diagnosis associated with the COVID-19 pandemic.

**Cancer**	**Appalachia (95% CI)**	**Non-Appalachia (95% CI)**	**P-value for Difference**
All HPV-associated	2.18 (1.75, 2.62)	1.40 (1.05, 1.74)	0.006
Oropharyngeal (female)	2.06 (0.47, 3.68)	0.70 (0.26, 1.14)	0.107
Oropharyngeal (male)	3.01 (2.36, 3.66)	2.35 (1.92, 2.79)	0.103
Anal (female)	5.12 (3.95, 6.30)	4.25 (3.86, 4.64)	0.133
Anal (male)	2.73 (0.17, 5.35)	2.70 (2.10, 3.31)	0.986
Vulvar	-0.43 (-1.68, 0.83)	-0.47 (-1.06, 0.12)	0.949
Vaginal	1.04 (-0.51, 2.61)	-0.35 (-1.10, 0.41)	0.086
Cervical	0.43 (0.00, 0.87)	-0.05 (-0.50, 0.40)	0.115
Penile	4.18 (2.54, 5.84)	1.77 (1.07, 2.48)	0.004

Abbreviations: CI = Confidence interval

© 2025 Burus T et al. *JAMA Network Open.*

eTable 5. Appalachian Human Papillomavirus (HPV)-Associated Cancer Incidence Rates by Urbanicity, 2017-2021, US Cancer Statistics[5]

**Cancer**	**Nonmetropolitan (95% CI)**	**Metropolitan (95% CI)**	**Rate ratio, ref. Metropolitan (95% CI)**
All HPV-associated	15.35 (15.01, 15.70)	13.83 (13.60, 14.07)	1.11 (1.08, 1.14)
Oropharyngeal (female)	2.24 (2.08, 2.43)	1.93 (1.82, 2.05)	1.16 (1.12, 1.21)
Oropharyngeal (male)	10.33 (9.96, 10.71)	10.04 (9.77, 10.31)	1.03 (0.98, 1.08)
Anal (female)	3.14 (2.93, 3.36)	2.87 (2.73, 3.02)	1.09 (1.00, 1.19)
Anal (male)	1.31 (1.17, 1.46)	1.35 (1.25, 1.46)	0.97 (0.85, 1.11)
Vulvar	3.37 (3.16, 3.60)	2.77 (2.63, 2.92)	1.22 (1.12, 1.32)
Vaginal	0.48 (0.40, 0.57)	0.44 (0.38, 0.50)	1.10 (0.89, 1.37)
Cervical	8.67 (8.26, 9.09)	7.40 (7.14, 7.67)	1.17 (1.10, 1.24)
Penile	1.25 (1.11, 1.39)	0.88 (0.79, 0.97)	1.42 (1.23, 1.65)

Abbreviations: CI = Confidence interval

© 2025 Burus T et al. *JAMA Network Open.*

eTable 6. Appalachian Human Papillomavirus (HPV)-Associated Cancer Incidence by Race and Ethnicity, 2017-2021, US Cancer Statistics[5]

**Cancer**	**Hispanic (95% CI)**	**NH Black (95% CI)**	**NH White (95% CI)**	**Other NH Races[a] (95% CI)**
All HPV-associated	7.92 (7.07, 8.85)	10.08 (9.53, 10.65)	15.15 (14.94, 15.37)	9.39 (8.31, 10.56)
Oropharyngeal (female)	-	1.18 (0.94, 1.45)	2.20 (2.09, 2.31)	-
Oropharyngeal (male)	3.23 (2.46, 4.14)	6.00 (5.39, 6.65)	10.86 (10.61, 11.10)	5.23 (4.09, 6.57)
Anal (female)	2.00 (1.37, 2.79)	1.81 (1.51, 2.16)	3.18 (3.05, 3.32)	-
Anal (male)	-	1.53 (1.23, 1.89)	1.34 (1.25, 1.44)	-
Vulvar	1.22 (0.75, 1.84)	1.53 (1.25, 1.86)	3.20 (3.06, 3.34)	1.75 (1.14, 2.57)
Vaginal	-	0.55 (0.39, 0.75)	0.46 (0.41, 0.51)	-
Cervical	7.76 (6.68, 8.95)	6.61 (6.00, 7.26)	8.07 (7.82, 8.32)	7.42 (6.16, 8.86)
Penile	-	0.74 (0.52, 1.01)	1.02 (0.95, 1.11)	1.30 (0.71, 2.15)

[a] Other non-Hispanic races includes individuals identified as non-Hispanic American Indian/Alaska Native, non-Hispanic Asian or Pacific Islander, and non-Hispanic unknown race

(-) indicates that rates are suppressed due to having fewer than 16 cases diagnosed

Abbreviations: NH = non-Hispanic; CI = confidence interval

© 2025 Burus T et al. *JAMA Network Open.*

eTable 7. Trends in Human Papillomavirus (HPV)-Associated Cancer Incidence by Appalachian Subregion, 2004-2021, US Cancer Statistics[5]

Average annual percentage change (AAPC) between in incidence rates between 2004 and 2021 estimated by joinpoint regression. Rates from 2020 excluded due to disruptions in diagnosis associated with the COVID-19 pandemic.

Cancer	Northern (95% CI)	North Central (95% CI)	Central (95% CI)	South Central (95% CI)	Southern (95% CI)
All HPV-associated	1.79 (1.47, 2.12)	1.96 (1.34, 2.59)	1.94 (1.41, 2.46)	1.30 (-0.11. 2.74)	0.94 (0.04, 1.84)
Oropharyngeal (female)	1.68 (-0.52, 3.92)	2.23 (0.16, 4.34)	-	2.05 (0.84, 3.28)	0.34 (-0.70, 1.39)
Oropharyngeal (male)	3.38 (2.84, 3.93)	3.88 (2.99, 4.77)	3.20 (2.13, 4.29)	2.45 (1.21, 3.70)	1.47 (0.43, 2.52)
Anal (female)	3.81 (2.60, 5.04)	3.99 (2.37, 5.64)	2.21 (0.24, 4.23)	2.86 (1.53, 4.20)	1.62 (0.34, 2.91)
Anal (male)	2.27 (0.89, 3.67)	-	-	0.91 (-3.54, 5.56)	1.23 (-0.02, 2.11)
Vulvar	1.79 (0.87, 2.72)	2.71 (1.76, 3.67)	2.47 (1.17, 3.78)	3.61 (-0.27, 7.64)	1.01 (0.15, 1.88)
Vaginal	0.34 (-2.21, 2.96)	-	-	-	-
Cervical	-0.14 (-0.66, 0.39)	-0.64 (-1.75, 0.48)	0.71 (-0.40, 1.83)	-0.38 (-1.13, 0.38)	-0.12 (-0.76, 0.52)
Penile	1.03 (-1.04, 3.14)	-	-	0.96 (-1.27, 3.25)	2.30 (0.39, 4.24)

(-) indicates that rates are suppressed due to having fewer than 16 cases diagnosed

Abbreviations: CI = confidence interval

© 2025 Burus T et al. *JAMA Network Open.*

eTable 8. Trends in Late-Stage Human Papillomavirus (HPV)-Associated Cancer Incidence by Appalachian Subregion, 2004-2021, US Cancer Statistics[5]

Average annual percentage change (AAPC) between in incidence rates between 2004 and 2021 estimated by joinpoint regression. Rates from 2020 excluded due to disruptions in diagnosis associated with the COVID-19 pandemic.

**Cancer**	**Northern (95% CI)**	**North Central (95% CI)**	**Central (95% CI)**	**South Central (95% CI)**	**Southern (95% CI)**
All HPV-associated	0.79 (0.03, 1.56)	1.17 (0.11, 2.25)	0.78 (-0.38, 1.96)	0.14 (-0.48, 0.77)	0.42 (-0.17, 1.02)
Oropharyngeal (female)	1.47 (-1.56, 4.60)	-	-	2.35 (0.98, 3.74)	0.48 (-0.97, 1.95)
Oropharyngeal (male)	3.89 (2.84, 4.95)	3.36 (1.58, 5.17)	3.99 (2.93, 5.06)	3.03 (1.54, 4.55)	1.38 (-1.50, 4.35)
Anal (female)	6.23 (4.58, 7.90)	-	-	-	2.61 (0.68, 4.58)
Anal (male)	-	-	-	-	-
Vulvar	-0.11 (-1.75, 1.55)	-	-	-0.47 (-2.94, 2.06)	-1.34 (-3.26, 0.61)
Vaginal	-	-	-	-	-
Cervical	0.53 (-0.24, 1.30)	-0.05 (-1.44, 1.36)	1.70 (0.23, 3.18)	0.47 (-0.70, 1.65)	0.10 (-0.91, 1.12)
Penile	-	-	-	-	-

(-) indicates that rates are suppressed due to having fewer than 16 cases diagnosed

Abbreviations: CI = confidence interval

© 2025 Burus T et al. *JAMA Network Open.*

eFigure 2. Trends in Late-Stage Cervical Carcinoma Incidence Rates in Central Appalachia, 2004-2021, US Cancer Statistics[5]

Incidence rates for 2004-2021 with fitted joinpoint regression model and segment annual percentage change shown. Rates for 2020 were excluded from models due to the impact of the COVID-19 pandemic on cancer diagnoses. Comparison made to trends in non-Appalachia. Significant annual percentage change indicated by (*).

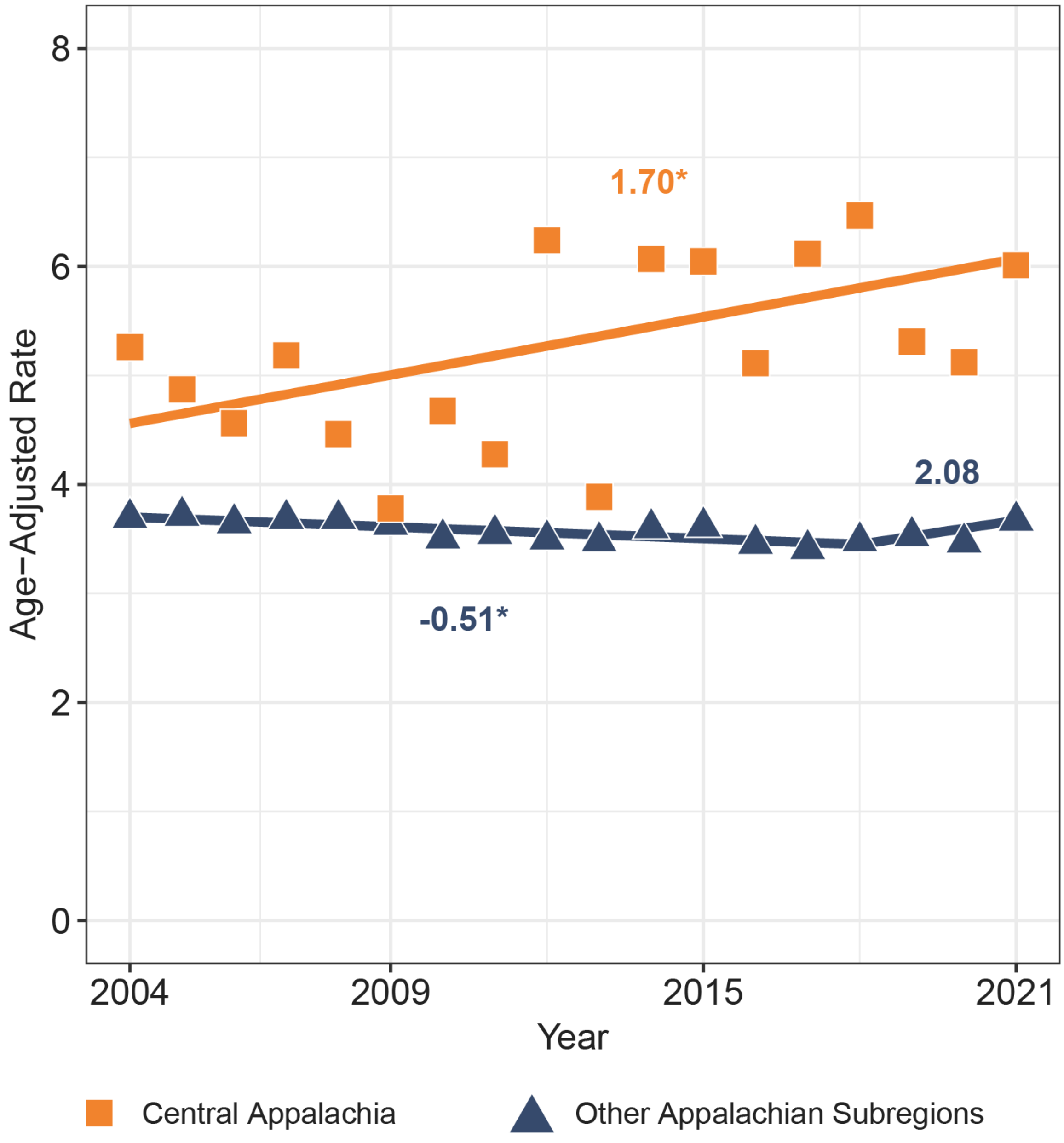

© 2025 Burus T et al. *JAMA Network Open.*

eReferences.

1. United States Cancer Statistics. Definitions of Risk Factor-Associated Cancers. U.S. Centers for Disease Control and Prevention. June 13, 2024. Accessed October 1, 2024. https://www.cdc.gov/united-states-cancer-statistics/public-use/definitions-risk-factor-associated-cancers.html

2. Fritz AG, ed. *International Classification of Diseases for Oncology: ICD-O*. 3rd ed. World Health Organization; 2000.

3. Appalachian Regional Commission. Accessed March 15, 2024. https://www.arc.gov/

4. U.S. Census Bureau. American Community Survey 5-Year Data (2018-2022). Published online 2023. Accessed August 24, 2024. https://www.census.gov/programs-surveys/acs

5. United States Department of Health and Human Services, Centers for Disease Control and Prevention. National Program of Cancer Registries SEER*Stat Database: USCS Incidence Analytic Database with single ages – 1998–2021 – linked to county attributes. Released June 2024, based on the 2023 submission.

© 2025 Burus T et al. *JAMA Network Open.*
