# Supplementary material for: Disparities in Human Papillomavirus–Associated Cancer Incidence by Appalachian Residence
Source: JAMA Netw Open. 2025 Jun 30;8(6):e2518242. doi: 10.1001/jamanetworkopen.2025.18242 (PMC12210082; doi:10.1001/jamanetworkopen.2025.18242)
Supplement: Supplement 2. — Data Sharing Statement [file jamanetwopen-e2518242-s002.pdf]

Data Sharing Statement

Burus. Disparities in Human Papillomavirus–Associated Cancer Incidence by Appalachian Residence. *JAMA Netw Open*. Published June 30, 2025.
doi:10.1001/jamanetworkopen.2025.18242

Data
Data available: Yes
Data types: Deidentified participant data
How to access data: Data used for this study were obtained via a restricted-use agreement with the Centers for Disease Control and Prevention's US Cancer Statistics. Aggregated and appropriately suppressed data pertaining to this study may be obtained from the study authors upon request (tburus@uky.edu).
When available: With publication

Supporting Documents
Document types: None

Additional Information
Who can access the data: Anyone
Types of analyses: For any purpose
Mechanisms of data availability: Reasonable request
Any additional restrictions: n/a
